# Serum melatonin concentration in critically ill patients randomized to sedation or non-sedation

**DOI:** 10.1186/s13613-021-00829-1

**Published:** 2021-03-06

**Authors:** Jakob Oxlund, Torben Knudsen, Thomas Strøm, Jørgen T. Lauridsen, Poul J. Jennum, Palle Toft

**Affiliations:** 1 Department of Anesthesiology and Intensive Care, Hospital of Southwest Jutland, Finsensgade 35, 6700 Esbjerg, Denmark; 2 Department of Internal Medicine, Hospital of Southwest Jutland, Finsensgade 35, 6700 Esbjerg, Denmark; 3 Department of Anesthesiology and Intensive Care, Odense University Hospital, J.B. Winsløwsvej 4, 5000 Odense, Denmark; 4 Department of Business and Economics, University of Southern Denmark, Campusvej 55, 5230 Odense, Denmark; 5 Department of Neurophysiology, Danish Center of Sleep Medicine. Rigshospitalet, Valdemar Hansens vej 1 – 23, 2600 Glostrup, Denmark

**Keywords:** Melatonin, Circadian, Rhythm, Sedation, Non-sedation, Delirium

## Abstract

**Background:**

Abolished circadian rhythm is associated with altered cognitive function, delirium, and as a result increased mortality in critically ill patients, especially in those who are mechanically ventilated. The causes are multifactorial, of which changes in circadian rhythmicity may play a role. Melatonin plays a crucial role as part of the circadian and sleep/wake cycle. Whether sedation effects circadian regulation is unknown. Hence, the objective of this study was to evaluate the melatonin concentration in critically ill patients randomized to sedation or non-sedation and to investigate the correlation with delirium.

**Methods:**

All patients were included and randomized at the intensive care unit at the hospital of southwest Jutland, Denmark. Seventy-nine patients completed the study (41 sedated and 38 non-sedated). S-melatonin was measured 3 times per day, (03.00, 14.00, and 22.00), for 4 consecutive days in total, starting on the second day upon randomization/intubation. The study was conducted as a sub-study to the NON-SEDA study in which one hundred consecutive patients were randomized to sedation or non-sedation with a daily wake-up call (50 in each arm). Primary outcome: melatonin concentration in sedated vs. non-sedated patients (analyzed using linear regression). Secondary outcome: risk of developing delirium or non-medically induced (NMI) coma in sedated vs. non-sedated patients, assessed by CAM-ICU (Confusion Assessment Method for the Intensive Care Unit) analyzed using logistic regression.

**Results:**

Melatonin concentration was suppressed in sedated patients compared to the non-sedated. All patients experienced an elevated peak melatonin level early on in the course of their critical illness (*p* = 0.01). The risk of delirium or coma (NMI) was significantly lower in the non-sedated group (OR 0.42 CI 0.27; 0.66 *p* < 0.0001). No significant relationship between delirium development and suppressed melatonin concentration was established in this study (OR 1.004 *p* = 0.29 95% CI 0.997; 1.010).

**Conclusion:**

Melatonin concentration was suppressed in sedated, critically ill patients, when compared to non-sedated controls and the frequency of delirium was elevated in sedated patients.

*Trail registration* Clinicaltrials.gov (NCT01967680) on October 23, 2013.

## Introduction

Sleeplessness and sleep fragmentation/disruption often lead to delirium, and are associated with increased mortality and are a frequent complication in the critically ill patient [1–4]. Typically, the patient presents with fragmented sleep with multiple episodes of interruptions in short intervals [5, 6]. The underlying causes are multifactorial, but comorbidities, primary illness, pain, medical treatment/sedation, alarms, lighting, sepsis, and mechanical ventilation are important factors [1, 6, 7]. Melatonin is considered one of the most important markers of circadian rhythm [7–9]. The suprachiasmatic nucleus located in the hypothalamus regulates the production of endogenous melatonin from the pineal gland via a cervical loop involving the cervical sympathetic ganglion [10]. Melatonin plays multiple physiological roles in the regulation of the sleep/wake cycle and bodily circadian entrainment by acting as an internal 24-h biological clock [6]. Strong light suppresses the production of endogenous melatonin, which in healthy adults usually starts increasing when daylight disappears around 20.00. Peak values are measured at approximately 04.00 after which the value drops steadily to baseline level at 12 noon [10].

Melatonin exerts its sleep facilitating effects through activation of MT1 and MT2 melatonin receptors. Suppression of neuronal activity by melatonin is one of the possible mechanisms by which it contributes to the regulations of sleep [10, 11].

Reduced melatonin concentration has been associated with a number of brain diseases, with delirium as an important and dominant manifestation in ICU (Intensive Care Unit) patients. Delirium has been associated with decreased levels of melatonin, although causality has not yet been firmly established in the literature. There are, however, potential strong associations between critical illness, circadian disruption, sleep disturbance, poor sleep quality, reduced cognitive function, and delirium [1, 3, 7].

## Materials and methods

### Objective

The effect of sedation on melatonin concentration has never been studied in a randomized trial and previous studies are often based on urine or saliva as surrogate measurement of serum melatonin [1, 7, 12]. Hence, the objective of this study was to evaluate the serum melatonin concentration in critically ill patients, and to investigate the risk of developing delirium or non-medically induced (NMI) coma and its correlation with suppressed melatonin concentration; particularly to investigate the effect of sedation on the melatonin concentration.

### Hypothesis

(1) Sedation suppresses the melatonin concentration compared to a non-sedation strategy. (2) The risk of developing delirium or coma (NMI) is increased in the sedated critically ill patient. (3) Suppressed melatonin concentration or decreased nocturnal melatonin peak correlates to increased risk of developing delirium or coma (NMI) in mechanically ventilated, critically ill patients.

### Trial design

This randomized prospective controlled single-center trial was conducted as a sub-study of the NON-SEDA study in which critically ill patients with a 1:1 allocation were randomized into parallel groups receiving either sedation or non-sedation with a daily wake-up trial. This study design allowed group equalization and comparison with a standard or routine treatment (light sedation). Approval was obtained from the local Ethics committees (S-20130025) and the protocol of the parent study was registered with the clinicaltrials.gov (NCT01967680). Written informed consent was obtained from either the patient or their closest relative and from the patient’s general practitioner (during working hours) or the Medical Health Office. For further details, the protocol of the NON-SEDA study and full article has been published previously in trials and the New England Journal of Medicine [13, 14].

### Participants

The NON-SEDA study was performed partly at the Intensive Care Unit at the Hospital of Southwest Jutland (a 12-bed mixed medical/surgical department). One hundred patients randomized to non-sedation or sedation with a daily wake-up trial (50 in each arm) were included in the NON-SEDA and the sub-study. Seventy-nine patients completed the study (41 sedated and 38 non-sedated). Twenty-one patients were excluded from the study after randomization as a result of early extubation. Inclusion criteria: intubated, mechanically ventilated patients aged 18 years and over, with an expected duration of mechanical ventilation > 24 h. Exclusion criteria: patients with severe head trauma, coma (non-medically induced) or status epilepticus upon admission, patients treated with therapeutic hypothermia, and patients with a ratio of the partial pressure of arterial oxygen (measured in kilopascal) to the fraction of inspired oxygen of 9 or lower. Patients were also excluded if sedation was anticipated to be necessary to ensure sufficient oxygenation or placing the patient in a prone position.

### Interventions

The experimental intervention was non-sedation supplemented with pain management during mechanical ventilation. The control intervention was light sedation with a daily wake-up trial.

### Intervention group

Patients in the intervention group did not receive sedatives, but bolus doses of morphine were used as analgesia. They were awake, able to communicate with the staff and measures to establish a natural circadian rhythm were attempted. If sedation, despite non-pharmacological and pharmacological treatment, became necessary as a result of agitation or insufficient oxygenation, the patient was sedated in accordance with the control group. Patients randomized to non-sedation, but in need of sedation to accept intubation were sedated, with intervals of 6 h until non-sedation was administered successfully. Data were handled according to intension to treat principles except patients were excluded from the present study after randomization if they were extubated within 48 h (24 h into the sub-study period).

### Control group

Sedation of the control group was performed with a continuous infusion of propofol during the first day and midazolam the following 3 days, with a target RASS (Richmond agitation-sedation scale) of − 2 to − 3 (light-to-moderate sedation: briefly awakens to voice and when lightly sedated: eye contact) according to international guidelines [15, 16]. Wake-up trial was performed every day and patients were considered awake when able to perform three of the following four tasks: (1) able to open eyes when verbally instructed to (2) able to follow a target with the eyes, (3) squeeze hands upon request, and (4) able to stick out the tongue upon request [17]. Ventilator weaning was performed during and after the wake-up trial. Sedation was resumed at half the pre-wake-up dose after successful wake-up trial. Likewise, sedation was resumed during the wake-up trial if the patient was uncomfortable.

Both groups were treated with paracetamol and morphine as bolus doses to keep the patients comfortable and pain-free. When necessary, epidural catheters were used. At 14.00 and 22.00 during the 4 day study period, the patients were assessed for delirium using the CAM–ICU score (Confusion Assessment Method–Intensive Care Unit [18]). If patients tested positive, they were registered as delirious at that time. In some cases, patients were unable to be evaluated for various reasons; RASS less than − 3, patients were undergoing procedure or examination or not willing to participate at the time (Fig. 4).

If treatment was required, non-pharmacological measures were used first, and if insufficient, pharmacological treatment with haloperidol or olanzapine was considered. Target sedation level was ensured continuously and registered twice per day using RASS at 14.00 and 22.00 [15]. No patients received beta-blockers, etomidate, melatonin, caffeine, or other activating alkaloids. Patients were not wearing eye masks during the night, as this would not be in keeping with the main study protocol. Lights were turned off at 21.00 and window blinds were drawn. Noise was kept to a minimum, and if possible, the patients were admitted to a single room with signs informing the staff to be silent from 21.00 to 08.00.

Reference melatonin concentration as presented in Fig. 2 were derived from the literature describing human physiology. The reference level was based on a population of healthy individuals at the age interval of the participants in the present study and as such not directly comparable to a population of critically ill patients [6, 12, 19].

Serum melatonin concentration in both groups was measured. A blood sample was drawn from the arterial cannula three times per day at 14.00, 22.00, and 03.00 for 4 consecutive days, starting 24 h after randomization. Samples were drawn at the specified times to correspond to peak concentration of melatonin according to human physiology [6, 10, 12]. Example: if a patient was randomized at 13.00 on day 1 the first sample was drawn at 22.00 on day 2. Twelve samples were drawn in total if the patient remained intubated throughout the 4-day study period. If the patient was extubated during the study period, blood samples were no longer drawn, as the patient would no longer be treated according to protocol. If patients were extubated less than 48 h after randomization (24 h into the sub-study period), they were excluded from the study as too few blood samples were drawn.

Within 1 h after the blood sample was collected, it was centrifuged and the plasma frozen at − 80 degrees Celsius. This procedure ensured that the blood sample could be stored and used for 1 year. The samples were transported on dry ice to Unilabs (Nygaardsvej 32, 2100 Copenhagen), an external laboratory facility to be analyzed for melatonin. Analysis was performed using ELISA (Enzyme-Linked Immunosorbent Assay). ELISA kit from IBL international (RE54021).

### Outcomes

Primary: serum melatonin concentration in sedated vs. non-sedated critically ill patients. Secondary: (1) risk of developing delirium or coma (NMI) in sedated vs. non-sedated patients during the 4-day study period, assessed by CAM-ICU performed at 14.00 and 22.00.

### Sample size

The sub-study was subjected to a fixed number of patients, and because the expected effect size was unknown, it was not possible to do a useful power calculation.

### Randomization

Patients were randomized to one of the two groups once intubated and mechanical ventilation was started. The randomization was carried out centrally by a computer-generated allocation sequence with a variable block size, which was kept concealed from the investigators at the clinical sites. The allocation sequence was stratified by age (up to 65 years or older) and shock upon admission (systolic blood pressure < 70 mmHg or below). One hundred patients included in the main study were included in the present study.

### Blinding

Due to the nature of the trial interventions, it was not possible to blind the investigators and the staff at the clinical trial sites, nor the participants and their relatives. All other parties in the trial were blinded. The statistical analyses were conducted blinded.

### Statistical methods

For the primary outcome, melatonin concentrations in the two groups were calculated as means with 95% confidence intervals, based on a linear regression analysis. An unadjusted version without control variables, reporting simple means and their confidence intervals, was performed (not reported here). An adjusted version with control variables included, thus reporting adjusted means and confidence intervals, is further provided. As control variables, age, gender, height, weight, SAPS II (Simplified Acute Physiology Score), APACHE (Acute Physiology and Chronic Health Evaluation), and SOFA (Sequential Organ Failure Assessment) were mean adjusted and included in the regression together with a group indicator. Multiple samples in the same individual were accounted for by including an individual random effect in the linear regression (i.e., a linear mixed model). Given that regression analysis was used, adjustment for multiple comparison was guaranteed.

The secondary outcome risk of delirium or coma (NMI) was analyzed using logistic regression with the same control variables as the primary outcome, and odds ratios (OR) were obtained together with 95% confidence intervals and p values. The secondary outcome RASS score was analyzed using a linear regression approach similar to what was described above for the primary outcome. Patients with positive CAM-ICU or coma (RASS < − 3) were considered delirious.

For assessment of baseline characteristics, T and Chi-square test were applied to determine baseline characteristic equality, as the data was normal distributed. The Mann–Whitney test was used to determine differences in medication administration between groups.

## Results

**Fig. 1:**
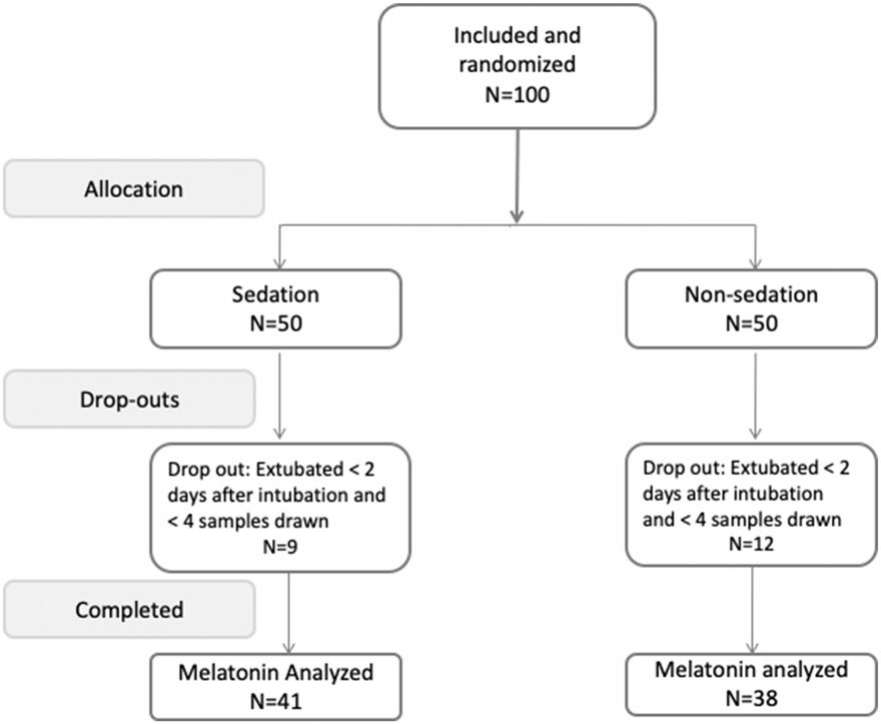
Consort

From September 2014 to December 2015, one hundred patients were randomized to sedation or non-sedation at the Intensive Care Unit at the Hospital of South West Jutland (50 patients in each arm). Seventy-nine patients completed the study. Thirty-eight patients were assigned to the intervention group receiving non-sedation and 41 patients were assigned to the control group receiving sedation with a daily wake-up trial. Twenty-one patients were excluded due to early extubation and less than 4 blood samples were drawn (Fig. 1).

The groups were similar with regards to weight, BMI (Body Mass Index), age, APACHE, SAPS II, and SOFA (day 1 to 4). In the sedated group, there were significantly more males and they were shorter than their non-sedated controls (Table 1).

**Table 1:** Baseline characteristics

	Non sedated *n* = 38 (48%)	Sedated *n* = 41(51%)	*p* value
Gender (female)	10 (26%)	20 (49%)	0.026
Height	173 (± 8)	170 (± 8)	0.056
Weight	85 (± 17)	81 (± 22)	Ns
BMI	28 (± 5)	28 (± 8)	Ns
Age	72 (± 10)	70 (± 11)	Ns
APACHE II	28 (± 7)	28 (± 8)	Ns
SAPS II	47 (± 17)	51 (± 13)	Ns
SOFA day 1	8 (± 4)	7 (± 4)	Ns
SOFA day 2	7 (± 4)	7 (± 5)	Ns
SOFA day 3	6 (± 3)	7 (± 4)	Ns
SOFA day 4	6 (± 3)	7 (± 4)	Ns
Respiratory insufficiency	17	17	
Sepsis	11	12	
Quinckes edema	1	0	
Influenza	0	1	
Pneumonia	3	6	
COPD	3	0	
Epiglottitis	0	1	
Rhabdomyolysis	1	0	

Data presented in Means with standard deviation. NS = nonsignificant (*p* > 0,1)

Italic values indicate primary value

### Primary outcome

**Fig. 2:**
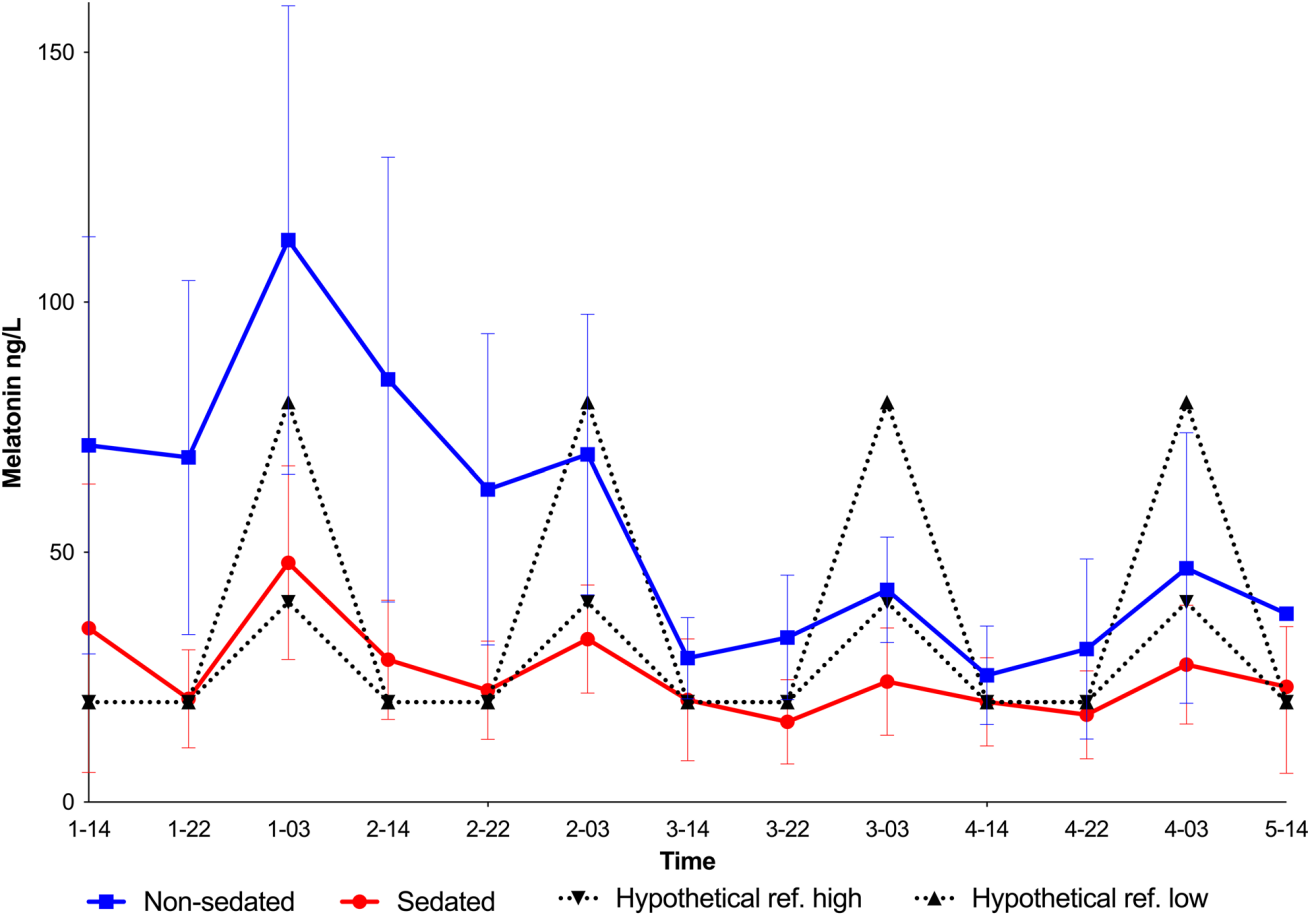
Mean melatonin concentration throughout the 4-day study period including 95% confidence interval. *X* axis; day and time of the sample (e.g., 2–14 = day 2 time 14:00). The reference curve is hypothetical and based on the literature, mainly M. Karasek et al. and demonstrates mean concentration of melatonin diurnal variation among healthy individuals in the age group of the study population [5, 12, 19]

Statistically significant differences in adjusted means of melatonin concentration at individual times were found on day 1 at 22.00 and 03.00 (*p* = 0.01), all three times on day 2 (*p* = 0.02) and at 22.00 and 03.00 on day 3 (*p* = 0.03 and *p* = 0.02) (Fig. 2). The number of patients in total in each group at individual times is presented in Table 2.

**Table 2:** Medication during the 4-day study period

	Day 1		*p*	Day 2		*p*	Day 3		*p*	Day 4		***p***
	SEDA	NON-SEDA		SEDA	NON-SEDA		SEDA	NON-SEDA		SEDA	NON-SEDA	
Propofol mg/kg/day	**12.59** 31/41 (0;34.5)	**0** 13/38 (0;2.17)	< 0,001	**1.77** 25/41 (0;16.3)	**0** 11/38 (0;0)	0.023	**0** 7/38 (0;0.3)	**0** 8/35 (0.72)	NS	**0** 4/34 (0;0)	**0** 8/31 0;1.53)	NS
Midazolam mg/kg/day	**0** 9/41 (0;0.68)	**0** 6/38 (0;0)	NS	**0.38** 33/41 (0;0.8)	**0** 6/38 (0;0)	< 0.001	**0.51** 30/38 (0.03;1.17)	**0** 5/35 (0;0)	< 0.001	**0.4** 24/34 (0;0.91)	**0** 4/35 (0;0)	< 0.001
Morphine mg/kg/day	**0.11** 34/41 (0.04;0.2)	**0.25** 35/38 (0.1;0.37)	0.007	**0.14** 38/41 (0.09;0.29)	**0.24** 34/38 (0.07;0.45)	NS	**0.13** 34/38 (0.05;0.26)	**0.15** 30/35 (0.06;0.54)	NS	**0.16** 31/34 (0.07;0.42)	**0.14** 26/31 (0.05;0.28)	NS
Clonidine μg/kg/day	**0** 4/41 (0;0)	**0** 13/38 (0;2.06)	0.002	**0** 9/41 (0;0.93)	**0** 14/38 (0;2.62)	NS	**0** 9/38 (0;1.09)	**0.69** 17/35 (0;3.5)	0,02	**0** 9/34 (0;0.71)	**2.21** 18/31 (0;3.94)	0.01
Halo-peridole mg/kg/day	**0** 1/41 (0;0)	**0** 7/38 (0;0)	0.02	**0** 1/41 (0;0)	**0** 6/38 (0;0)	0.04	**0** 2/38 (0;0)	**0** 7/35 (0;0)	0,08	**0** 0/34 (0;0)	**0** 7/31 (0;0)	0.09
Olanzapin mg/kg/day	**0** 1/41 (0;0)	**0** 0/38 (0;0)	NS	**0** 1/41 (0;0)	**0** 2/38 0;0	NS	**0** 0/38 (0;0)	**0** 5/35 (0;0)	0,009	**0** 0/34 (0;0)	**0** 5/31 (0;0)	0.016
Corticosteroid mg/kg/day	**0.2** 28/41 (0;1.6)	**0.36** 24/38 (0;2.15)	NS	**0.13** 25/41 (0;0.67)	**0.33** 24/38 (0;1.66)	NS	**0.11** 22/38 (0;0.67)	**0.26** 19/35 (0;0.73)	NS	**0** 15/34 (0;0.21)	**0** 13/31 (0;0.41)	NS
Patients N	**41**	**38**		**41**	**38**		**38**	**35**		**34**	**31**	

Data are presented in medians with Interquartile Range. Corticosteroids were administered as methylprednisolone, hydrocortisone and prednisolone and dosing was equalized. Actual number of patients receiving the medication along with total number of patients in the group at individual times has been presented. NS = *p* > 0.1. Patients *N* = Number of patients investigated at individual times

Mean melatonin elevation during the 4-day study period, when shifting from day to night (the nocturnal peak 14.00–03.00), in the sedated group was 5.9 ng/L (Interquartile Range (IQR) − 0.25;13.3) and in the non-sedated group 15.9 ng/L (IQR − 3;20.8, *p* > 0.1).

All patients experienced an elevated melatonin concentration during the first day compared to the last 2 days of the study period (*p* = 0.01, Fig. 2).

**Fig. 3:**
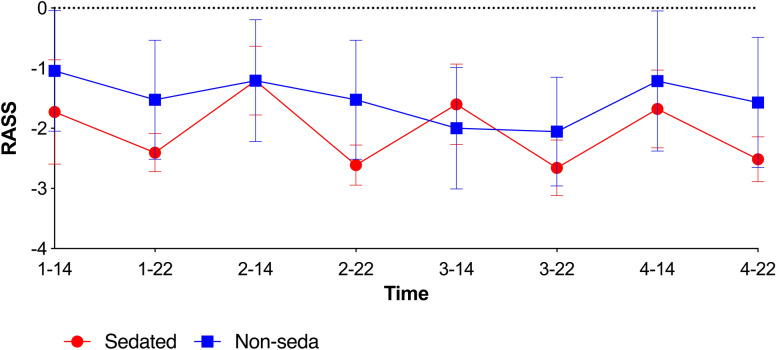
Mean RASS score throughout the 4-day study period including 95% confidence interval

The difference in adjusted RASS mean scores (Fig. 3) was 0.57 point higher for non-sedated compared to sedated patients (*p* = 0.001 95% CI 0.23;0.90). The difference at individual times during study period was not significant.

### Secondary outcomes

**Fig. 4:**
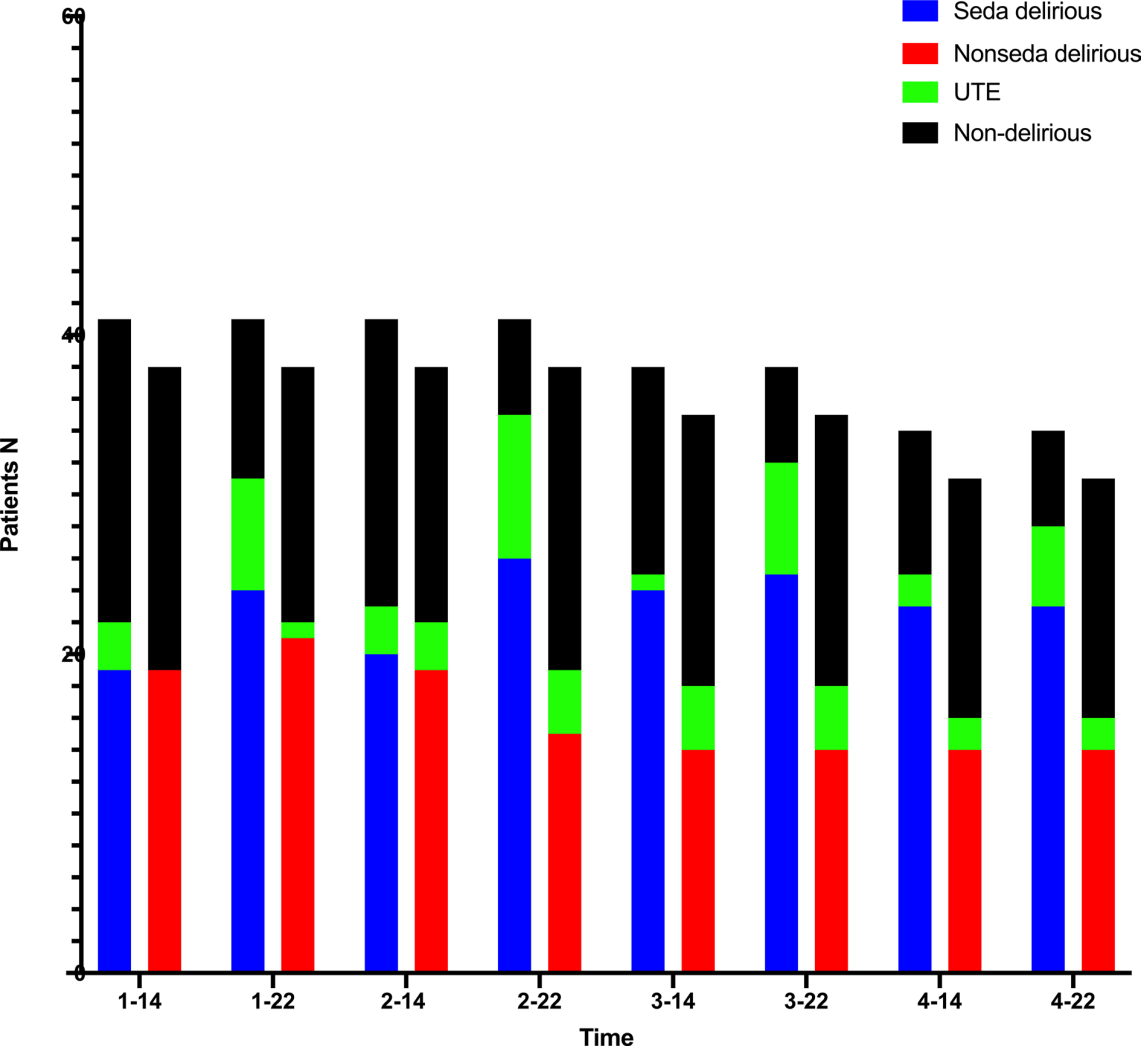
Patients *N* with positive and negative CAM-ICU test evaluated twice per day. UTE; Unable To Evaluate

The risk of developing delirium or coma (NMI) during the study period was significantly lower in the non-sedated group compared to the sedated (OR 0.42 95% CI 0.27; 0.66 *p* < 0.0001), based on CAM-ICU scores performed twice per day (14.00 and 22.00, Fig. 4). Patients were considered delirious at the time if they had an RASS score lower than – 3 or if they tested positive on the CAM-ICU test conducted as closely to 14.00 and 22.00 as could be achieved (Fig. 4). No significant effect of suppressed (decreased) melatonin concentration when comparing groups (OR 1,004 95% CI 0.997;1.010 *p* = 0,29) or decreased nocturnal melatonin peak value (delta melatonin concentration when shifting from day to night, estimated for each patient, each day and compared by group) on the risk of developing delirium or coma (NMI) was established in this study. When excluding comatose patients (RASS < − 3) from this equation, the risk of developing delirium during the study period was still insignificant (OR 1,008 95% CI 0.999; 1.018 *p* = 0.09).

Significantly more propofol and midazolam was administered in the sedated group according to the intervention protocol. The non-sedated group received significantly more rescue medication at specific times during the study period (i.e., morphine, haloperidol, and olanzapine). Corticosteroid was administered with no significant difference between the two groups; however, higher doses was administered in both groups during the first days of the study period.

## Discussion

The present randomized study reports that when investigating critically ill patients, randomized to sedation or non-sedation, melatonin concentration in serum was suppressed in sedated patients. No previous studies have evaluated the melatonin concentration in patients randomized to sedation vs. non-sedation.

Three modalities (blood, urine, and saliva) have been used to measure human melatonin, whereas the results of previous studies often are based on saliva or urine (aMT6s, melatonin metabolite). These modalities are associated with great advantages in a non-intensive care setting where, e.g., liver or renal failure is not an issue and where vascular access is not obtained [12, 20]. Previous studies present conflicting results regarding the effect of sedations on melatonin. Possibly for a number of reasons, e.g., measure modality, few patients enrolled, lack of randomized study design, and non-critically ill participants [6, 9, 21–24].

Mundigler et al. studied urine samples in septic critically ill patients sedated with midazolam and reported missing nocturnal elevation in melatonin secretion when compared to non-septic critically ill patients [9]. This is in keeping with the present findings, studying a population with a similar distribution of sepsis cases between the two groups (Table 2).

Previous studies investigating the effect of sepsis on melatonin levels in the critically ill patient are often conducted with no regard to pharmacological sedation and reporting conflicting results. Abolished circadian melatonin rhythmicity has been reported as well as decreased or elevated concentration in the septic critically ill patient [6, 20, 21, 25]. Because of melatonin’s immunomodulatory, antioxidant and antiapoptotic properties, it has been suggested that melatonin may play a role in the prevention of septic shock [5, 10].

In the present study, delirium or coma (NMI) was more frequent, melatonin concentration were suppressed, and melatonin fluctuations when shifting from day to night were decreased, in the sedated group. This or the previous studies do not provide evidence of a causal correlation/association between delirium and suppressed/decreased nocturnal melatonin peak value; however, Dessap et al. reported a significantly reduced nocturnal melatonin peak concentration, mean concentration, amplitude, and total 24-h excretion of aMT6s in patients suffering from delirium and these patients were more likely to be treated with propofol and midazolam [1].

Patients from both groups experienced elevated concentration of melatonin during the first days of their intensive care treatment. Previous studies suggest elevated melatonin concentration in blood and urine during critical illness in patients needing acute treatment, corticosteroids, and catecholamines [20, 21]. This treatment is often most pronounced at the beginning of an intensive care treatment. In the present study, more corticosteroid was administered in both groups during the first day of the study period (no significant dose difference between groups). Elevation of the stress hormone cortisol during the first days of critical illness has been described. However, cortisol concentration during a prolonged ICU admission often decreases and becomes inadequate [26, 27]. Since patients where shifted from propofol to midazolam on day 2, one could speculate that the decrease in melatonin concentration on day 3 and 4 was caused by the sedation change. However, the drop occurs in both groups.

In order for patients to stay awake and accept intubation more morphine was administered on day 1 in the non-sedated group. No significant dose difference was reported during the last 3 days and the suppressed melatonin concentration persisted. No significant difference when comparing melatonin concentration in patients receiving propofol and midazolam were found. The elevated melatonin concentration during the first days of the study period could in part be explained by the higher doses of morphine and corticosteroid during the first days in both groups [20, 21, 28].

The concentration of circulating hormones in the critically ill patients are often abnormal. The underlying causes are most likely multifactorial. It is likely that sedation has implications on a broad range of hormones [27]. Apart from cortisol, thyroid hormones are globally decreased in a prolonged intensive care admission, likely due to the restriction in thyrotropin, which significantly loses its pulsatile release [27]. Administration of growth hormone in the critically ill patient has been studied and shown to be potentially harmful [29]. The acute phase of all critical illnesses is characterized by major catecholaminergic activation, and it is well known that the use of most analgesic and sedative agents contributes to a decreased catecholamine decreasing. This is also one possible explanation for the suppressed melatonin concentration reported in the sedated group [20, 27]. Melatonin when studied with no reference to sedation often shows decreased concentrations [6, 7]. Hyposecretion of melatonin has potential serious clinical consequences, considering that melatonin has a wide range of physiological functions: association with an immunoenhancing effect has been established [30]. Prevention of acute gastric lesions induced by stress [31]. Impaired mitochondrial oxidative phosphorylation and the body’s ability to cope with endotoxemia have been described as likely effects of a normal melatonin secretion [32]. Furthermore, melatonin has cardioprotective properties via direct free radical activity [33].

Adjusting for SAPS II, APACHE and SOFA in the multiple regressions may potentially be redundant and there is a risk over adjusting. However, we found that results were comparable with and without these adjustments (the latter not reported here) and considered the fully adjusted results to be the most accurate. Furthermore, the linear regression approach was beneficial, as it adjusted for multiplicity of comparisons. Such multiplicity is occasionally handled using Bland–Altman diagrams; however, these do not allow adjustment for individual characteristics.

### Clinical perspective

Suppressed melatonin concentration in sedated patients as the present study reports adds to the current understanding of the consequences of sedation on circadian regulation. Further studies are needed to evaluate the correlation between melatonin and delirium and the use of melatonin as a possible marker for delirium and/or the potential benefit of melatonin administration to selected patients.

### Limitations

Melatonin analysis was for various reasons in the present study only performed three times per day during the first 4 days. In an optimal setting, the study period would have been longer, with more samples and included s-cortisol. For ethical reasons, the control group was subjected to light sedation as opposed to deep sedation. In turn, this created similar groups, thus reducing the possibility of reporting the full effect of sedation. Furthermore, 21 patients were excluded after randomization.

## Conclusion

The melatonin concentration was significantly decreased in sedated patients compared to non-sedated. All patients experienced a higher peak melatonin level early in the course of their critical illness. The risk of delirium or non-medically induced coma was significantly increased in the sedated group. No correlation between delirium development and suppressed melatonin level was found.

## Data Availability

The dataset used and analyzed during the current study are available from the corresponding author on reasonable request.

## Abbreviations

CAM-ICU: Confusion assessment method for the intensive care unit
ICU: Intensive care unit
RASS: Richmond agitation scale score
ELISA: Enzyme-linked immunosorbent assay
SAPS II: Simplified acute physiology score
APACHE: Acute physiology and chronic health evaluation
SOFA: Sequential organ failure assessment
IQR: Interquartile range
OD: Odds ratio
CI: Confidence interval
